# The diagnostic potential of oxidative stress biomarkers for preeclampsia: systematic review and meta-analysis

**DOI:** 10.1186/s13293-022-00436-0

**Published:** 2022-06-04

**Authors:** Dinara Afrose, Hao Chen, Amali Ranashinghe, Chia-chi Liu, Annemarie Henessy, Philip M. Hansbro, Lana McClements

**Affiliations:** 1 School of Life Sciences & Institute for Biomedical Materials and Devices (IBMD), Faculty of Science, University of Technology Sydney, Ultimo, NSW 2007 Australia; 2 Centre for Inflammation, Centenary Institute, and University of Technology Sydney, Faculty of Science, Sydney, NSW 2050 Australia; 3 Faculty of Science, University of Colombo, Colombo 03, Sri Lanka; 4 The Heart Research Institute, University of Sydney, Newtown, NSW Australia; 5 School of Medicine, Western Sydney University, Campbelltown, Australia; 6 Campbelltown Hospital, South Western Sydney Local Health District, Warwick Farm, Australia

**Keywords:** Preeclampsia, Oxidative stress, Heavy metals, Biomarkers, MDA, IMA, Uric acid

## Abstract

**Background:**

Preeclampsia is a multifactorial cardiovascular disorder of pregnancy. If left untreated, it can lead to severe maternal and fetal outcomes. Hence, timely diagnosis and management of preeclampsia are extremely important. Biomarkers of oxidative stress are associated with the pathogenesis of preeclampsia and therefore could be indicative of evolving preeclampsia and utilized for timely diagnosis. In this study, we conducted a systematic review and meta-analysis to determine the most reliable oxidative stress biomarkers in preeclampsia, based on their diagnostic sensitivities and specificities as well as their positive and negative predictive values.

**Methods:**

A systematic search using PubMed, ScienceDirect, ResearchGate, and PLOS databases (1900 to March 2021) identified nine relevant studies including a total of 343 women with preeclampsia and 354 normotensive controls.

**Results:**

Ischemia-modified albumin (IMA), uric acid (UA), and malondialdehyde (MDA) were associated with 3.38 (95% CI 2.23, 4.53), 3.05 (95% CI 2.39, 3.71), and 2.37 (95% CI 1.03, 3.70) odds ratios for preeclampsia diagnosis, respectively. The IMA showed the most promising diagnostic potential with the positive predictive ratio (PPV) of 0.852 (95% CI 0.728, 0.929) and negative predictive ratio (NPV) of 0.811 (95% CI 0.683, 0.890) for preeclampsia. Minor between-study heterogeneity was reported for these biomarkers (Higgins’ *I*^2^ = 0–15.879%).

**Conclusions:**

This systematic review and meta-analysis identified IMA, UA, and MDA as the most promising oxidative stress biomarkers associated with established preeclampsia. IMA as a biomarker of tissue damage exhibited the best diagnostic test accuracy. Thus, these oxidative stress biomarkers should be further explored in larger cohorts for preeclampsia diagnosis.

**Graphical Abstract:**

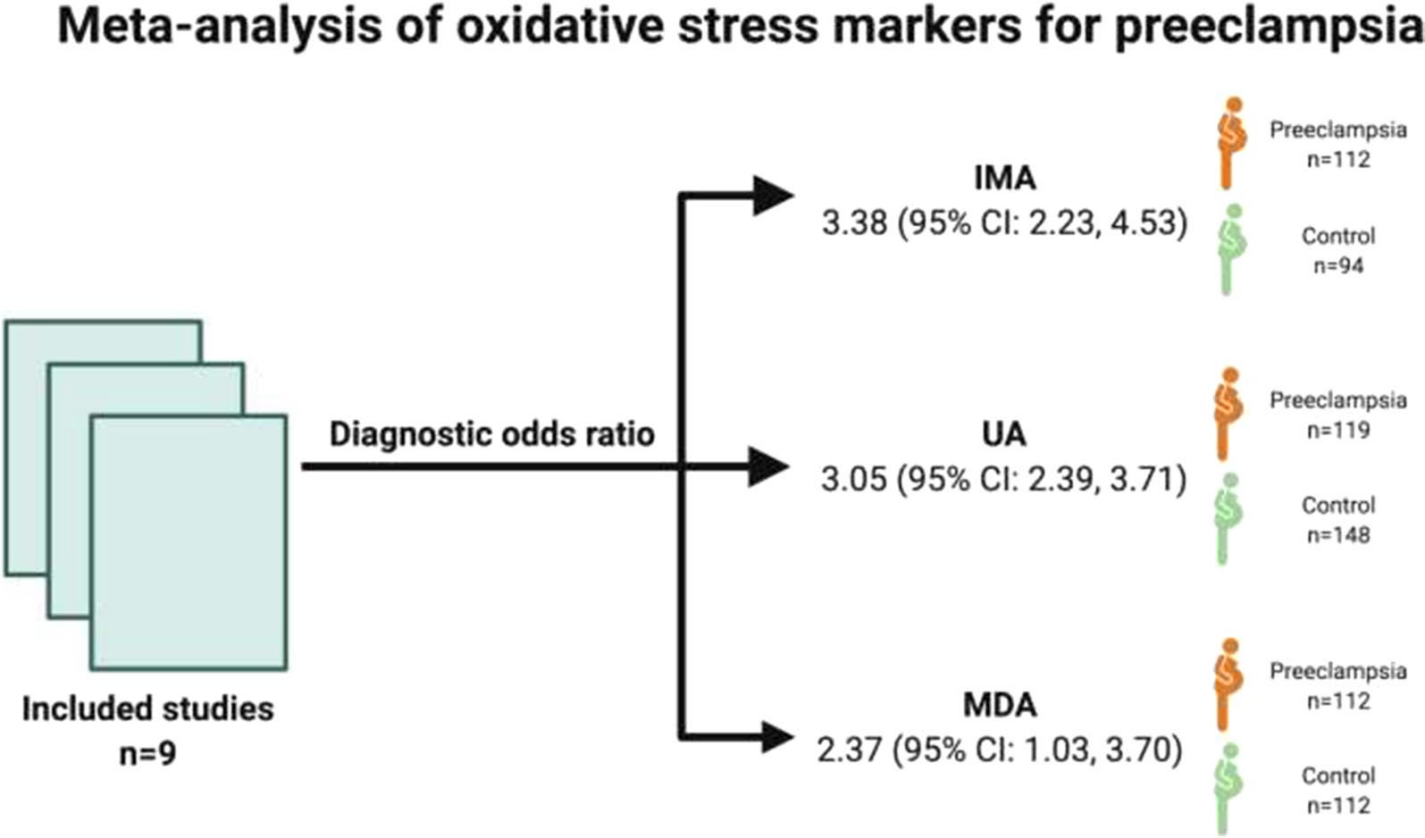

**Supplementary Information:**

The online version contains supplementary material available at 10.1186/s13293-022-00436-0.

## Highlights

- Biomarkers of oxidative stress are related to the pathogenesis of preeclampsia and might be indicative of evolving preeclampsia and utilized for timely diagnosis and management of preeclampsia.
- Systematic review and meta-analysis were conducted to evaluate the diagnostic accuracy of oxidative stress markers based on their diagnostic sensitivities and specificities.
- Clinically relevant positive predictive values (PPVs) and negative predictive values (NPVs) were determined for each biomarker.
- IMA, UA, and MDA were associated with 3.38, 3.05, and 2.37 odds ratios for preeclampsia onset.
- IMA exhibited the most promising diagnostic potential with an average PPV of 0.852 and NPV of 0.811, respectively. Minor heterogeneity was reported for these biomarkers (Higgins’ *I*^2^ = 0–15.879%).
- These oxidative stress markers should be further explored in larger cohorts for preeclampsia diagnosis.

**Supplementary Information:**

The online version contains supplementary material available at 10.1186/s13293-022-00436-0.

## Background

Preeclampsia is a multifactorial hypertensive disorder of the second half of pregnancy. It affects approximately 5–10% of pregnancies and contributes to 15% of all maternal deaths as well as between 275,000 and 1,375,000 fetal deaths worldwide each year [1]. It is diagnosed in women with a new-onset of hypertension (≥ 140/90 mmHg) and proteinuria (≥ 300 mg proteins in 24 h) or other organ dysfunction including in the kidneys, liver, or cerebrovascular system [2–5]. Some countries have reported that ~ 10 to 25% of preeclampsia cases result in maternal death [6, 7]. A plethora of studies have been conducted to elucidate the pathogenic mechanisms of preeclampsia, however early and reliable diagnostic strategies are still lacking, with the delivery of the baby and placenta being the only definitive treatment [3, 8, 9]. Late preeclampsia diagnosis can lead to severe complications including eclampsia, hemolysis, elevated liver enzymes, low platelet count (HELLP) syndrome, cerebral hemorrhage, thrombocytopenia, and other end-organ impairment [10]. Thus, the timely diagnosis could significantly improve the clinical management of preeclampsia, and further decrease associated morbidity and mortality [10, 11]. Using biomarkers in easily accessible clinical samples (e.g., blood, urine) can enhance the early diagnosis of any disease and provide insights into the mechanisms of its pathogenesis [12]. Various biological samples in pregnancy are utilized for determining the expression or concentration of certain diagnostic biomarkers including maternal blood/serum/plasma/urine or placental tissue [12]. Nevertheless, obtaining placental tissue during early pregnancy through chorionic villous sampling (CVS) is risky and can lead to miscarriage. Therefore, blood or urine-based biomarkers reflective of placental health could be very useful for identifying cases of evolving preeclampsia. In this systematic review and meta-analysis, the vast majority of the identified studies used serum/plasma [6, 13–18], whole blood/leukocytes [19–21], or placental tissue [22, 23] to quantify oxidative stress biomarkers related to preeclampsia diagnosis during or at the end of pregnancy.

Despite ongoing research focused on the pathophysiology of preeclampsia, its exact etiology remains incompletely understood, likely due to its multifactorial nature and different phenotypes. Mitochondrial dysfunction leading to oxidative stress of the placenta has been implicated as an early aberrant process in pregnancy, which results in the onset of preeclampsia [24]. Oxidative stress emerges when the balance between the levels of reactive oxygen species (ROS) exceeds the antioxidant capacity of a target cell [14]; this impaired balance during pregnancy can lead to aberrant placentation, which is the root cause of preeclampsia [25].

Recently, oxidative stress markers and trace metals have emerged as diagnostic biomarkers for preeclampsia. Abnormal levels of some trace metals including copper (Cu), zinc (Zn), or selenium (Se) are also associated with preeclampsia [5, 7, 11, 16, 19, 26]. Based on the key pathogenic role of oxidative stress in preeclampsia, several oxidative stress markers have been identified to have promising diagnostic potential [10]. The purpose of this systematic review and meta-analysis was to determine the most reliable oxidative stress and trace metals biomarkers that could aid in preeclampsia diagnosis and perhaps be explored as therapeutic targets in the future.

We identified three specific oxidative stress biomarkers [ischemia-modified albumin (IMA), uric acid (UA), malondialdehyde (MDA)], which exhibited reliable diagnostic odd ratio (DORs) indicative of their diagnostic potential in preeclampsia. These biomarkers have reliable pooled positive predictive values (PPVs) and negative predictive values (NPVs) alongside the sensitivities and specificities for the diagnosis of preeclampsia.

## Materials and methods

### Search strategy and selection criteria

The systematic search was conducted to assess the diagnostic reliability of oxidative stress markers and trace metals in preeclampsia using the following databases: PubMed, ScienceDirect, ResearchGate, and PLOS (1900 to March 2021). The literature search was performed using the following terms “oxidative stress markers” OR “heavy metals” AND “preeclampsia” OR “pre-eclampsia”. We included studies where preeclampsia was diagnosed as per American College of Obstetricians and Gynaecologists (ACOG) guidelines [27], which include high blood pressure (≥ 140/90 mmHg) with proteinuria (≥ 0.3 mg/24 h). In the absence of proteinuria, damage to other organs in addition to the new-onset hypertension including thrombocytopenia < 100.000/µL, liver transaminases more than twice that of normal values, HELLP syndrome, persistent epigastric or right upper quadrant pain, acute pulmonary edema, visual or neurological disorders, comprise preeclampsia diagnosis [28]. To determine the oxidative stress biomarkers’ suitability for preeclampsia diagnosis, published data from observational studies assessing the diagnostic accuracy of individual biomarkers to discriminate between groups with and without preeclampsia were included. Studies were selected if diagnostic performance measures of individual biomarkers were reported. Studies were excluded if: non-English language publications, conference abstracts, meta-analyses, reviews, letters, editorials, case reports, or animal studies.

### Data extraction

Data extraction was performed by two investigators (DA, HC). A third investigator (LM) resolved disagreements by consensus. The recommendations of PRISMA guidelines [29] and an appropriate guideline specific for biomarker meta-analysis [30] were followed for data extraction. A conventional 2 × 2 table comprising true positive (TP), true negative (TN), false positive (FP), and false negative (FN), values was extracted from individual studies. PPVs and NPVs was calculated based on TP, FP, TN and FN values. Only published data were extracted.

### Quality assessment

The included studies were evaluated for quality independently by two independent reviewers (DA and HC) utilizing the Quality Assessment for Diagnostic Accuracy Studies-2 (QUADAS-2) tool [31]. This assessment comprised four domains including (i) patient selection, (ii) index test, (iii) reference standard, and (iv) patient flow and timing. Low risk of bias in a domain referred to a total of one positive answer in sub-questions. High risk of bias in a domain referred to negative answers in two or three sub-questions. Unclear risk of bias was denoted as one or two negative answers. Results were compared between assessors and, in case of disagreement, individual studies were discussed to achieve consensus.

### Statistical analyses

The analyses of diagnostic test accuracy were performed in R (4.0.3) using the ‘mada’ package, where a bivariate, random-effects meta-analysis model was applied. The analyses of diagnostic biomarkers were based on sensitivity and specificity as well as the diagnostic odds ratio (DOR) to discriminate between women with and without preeclampsia. DOR is a measure of the effectiveness of a diagnostic test. Estimated diagnostic accuracy measures were calculated using the 2 × 2 tables extracted from the included studies. Sensitivity and specificity were pooled and analyzed to generate random-effects model forest plots and random-effects model hierarchical summaries of receiver operating characteristic (HSROC) curves. Natural logarithm (ln) transformed DOR was reported along with heterogeneity of Higgins’ *I*^2^ and Cochran’s *Q*. Publication bias was assessed through visual inspection of the funnel plots of ln(DOR). Analyses were generated only for those diagnostic markers, which were evaluated in three or more independent studies.

## Results

### Search results and study characteristics

A table with the study characteristics was generated for included studies (*n* = 9) evaluating oxidative stress markers for preeclampsia diagnosis in the meta-analysis (Table 1).

**Table 1:** Study characteristics of oxidative stress (OS) markers of included studies

Name of oxidative stress (OS) markers	Author/Reference	Study design	Sample	Optimal cut-off	Control type	*n* (Control)	PE type	*n* (PE)	GA at sampling	Exclusion criteria
Ischemia-modified albumin (IMA)	Ustun et al. [13]	Case–control	Serum	> 0.31 ABSU or ng/ml	Normotensive, non-smoker	18	EOPE and LOPE are mixed	36	32–38 weeks	Multiple pregnancy, chronic hypertension, renal disease, diabetes mellitus, other pre-existing disorders, the early history of preeclampsia, HELLP syndrome, immunosuppression, or a history of using illicit drugs
	Vyakaranam et al. [14]	Case–control	Serum	> 38.33 ng/mL	Normotensive	19	EOPE and LOPE are mixed	19	≥ 32 weeks	History of pregnancy complications, twin pregnancy, previous pregnancy with hypertensive disorders, pre-existing chronic conditions including diabetes mellitus, chronic hypertension, ischemic heart disease, peripheral vascular diseases
	Bambrana et al. [6]	Case–control	Serum	NA	Normotensive, non-smoker	57	NA	57	30–39 weeks before delivery and after delivery within 48 h	Pre-existing renal disease, thyroid disorders, chronic hypertensive disorder, gestational diabetes mellitus, epilepsy, hypertensive encephalopathy, heart disease, multiple pregnancies, fetal anatomical anomalies
Malondialdehyde (MDA)	Rani et al. [22]	Case–control	Placental tissue	6.5 nmol/g	Normotensive	30	EOPE and LOPE are mixed	30	Within 20 min of delivery	Chorioamnionitis, chronic hypertension, renal disease, cardiovascular disease, active asthma, thyroid disorders, and a history of seizures
	Bambrana et al. [6]	Case–control	Serum	NA	Normotensive, non-smoker	57	NA	57	30–39 weeks before delivery and after delivery within 48 h	Pre-existing renal disease, thyroid disorders, chronic hypertensive disorder, gestational diabetes mellitus, epilepsy, hypertensive encephalopathy, heart disease, numerous pregnancies, fetal anatomical anomalies
	Shaker et al. [23]	Case–control	Placental tissue	0.14 nmol/mg	Normotensive	25	EOPE and LOPE are mixed	25	Immediately after delivery	Pre-existing hypertension before 20 weeks gestation and women with pregnancy complications including diabetes mellitus, peripheral vascular disease, chronic renal disease, multifetal gestation, or major fetal anomalies
Uric acid (UA)	Nikolic et al. [3]	Case–control	Serum	> 242 μmol/L	Normotensive	60	EOPE and LOPE are mixed	32	≥ 24 weeks	Multifetal gestation, abnormal fetal morphology, pre-existing diseases, and gestational age prior to 24 weeks
	Bambrana et al. [6]	Case–control	Serum	NA	Normotensive, non-smoker	57	NA	57	30–39 weeks before delivery and after delivery within 48 h	Pre-existing renal disease, thyroid disorders, chronic hypertensive disorder, gestational diabetes, epilepsy, hypertensive encephalopathy, heart disease, numerous pregnancies, fetal anatomical anomalies
	Vyakaranam et al. [15]	Case–control	Serum	> 4.9 mg/dL	Normotensive	31	EOPE and LOPE are mixed	30	> 32 weeks	Repeated abortions, previous pregnancy complications, twin pregnancy, pre-existing medical disorders: diabetes mellitus, chronic hypertension, renal diseases, cardiovascular diseases, thyroid disorders, and liver disease

*PE* preeclampsia; *EOPE* early-onset preeclampsia; *LOPE* late-onset preeclampsia; *TP* true positive; *FP* false positive; *TN* true negative; *FN* false negative; *GA* gestational age; *HELLP syndrome* (hemolysis, elevated liver enzymes, and low platelet count) syndrome; *IUGR* intrauterine growth retardation; *GH* gestational hypertension; *PTB* pre-term birth

**Fig. 1:**
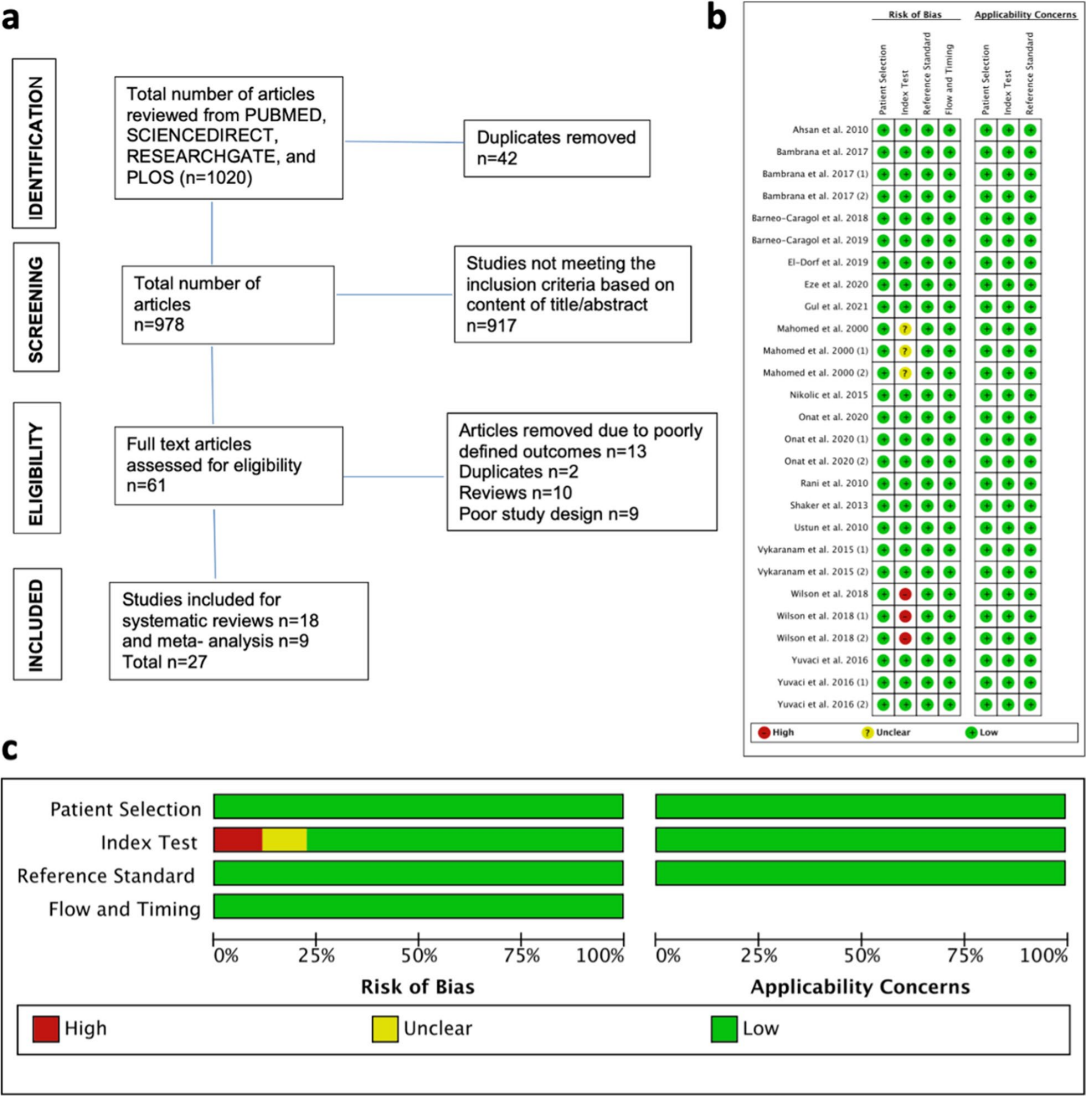
Study design of meta-analysis. **a** Preferred reporting items for systematic reviews and meta-analyses (PRISMA) guidelines flow diagram. **b** Risk of bias and applicability concerns summary. **c** Risk of bias and applicability concerns graphical presentation

Our search for oxidative stress markers and trace metals as diagnostic biomarkers for preeclampsia yielded a total of 1020 articles, of which 27 met the inclusion criteria (Fig. 1a). The vast majority of included studies were designed as case–control (*n* = 23) with some (*n* = 4) cohort studies [3, 5–7, 11, 13–23, 26]. The studies included (*n* = 9) in the meta-analyses reported that controls and women with preeclampsia were matched for maternal age. Related to other clinical parameters, only one study [23] reported parity, which was matched with enrolled healthy pregnant women. Mode of delivery was seldom reported in included studies, whereas gestational age at sampling is included in Table 1. Although the majority of studies (*n* = 24) measured biomarkers in blood or blood-derived samples [3, 5–7, 13–21, 26], placental tissues and urine samples were also used (*n* = 2) [22, 23] and (*n* = 1) [11]. The quality of the studies was satisfactory, eliminating the concern of bias and enhancing the robustness of this meta-analysis and systematic reviews.

### Oxidative stress biomarkers

Three oxidative stress biomarkers evaluated individually in at least three independent studies were selected for meta-analyses in this study. These were IMA, UA, malondialdehyde, all well-established oxidative stress markers. We were unable to complete meta-analyses for emerging biomarkers (disulfide, native thiol, total thiol, and strontium) due to a limited number of supporting references (*n* < 3 studies per biomarker). In the majority of included studies, preeclampsia was not stratified into different phenotypes, namely early-onset or late-onset preeclampsia. All women with preeclampsia were free from chronic hypertension, chronic renal diseases, and diabetes mellitus.

**Fig. 2:**
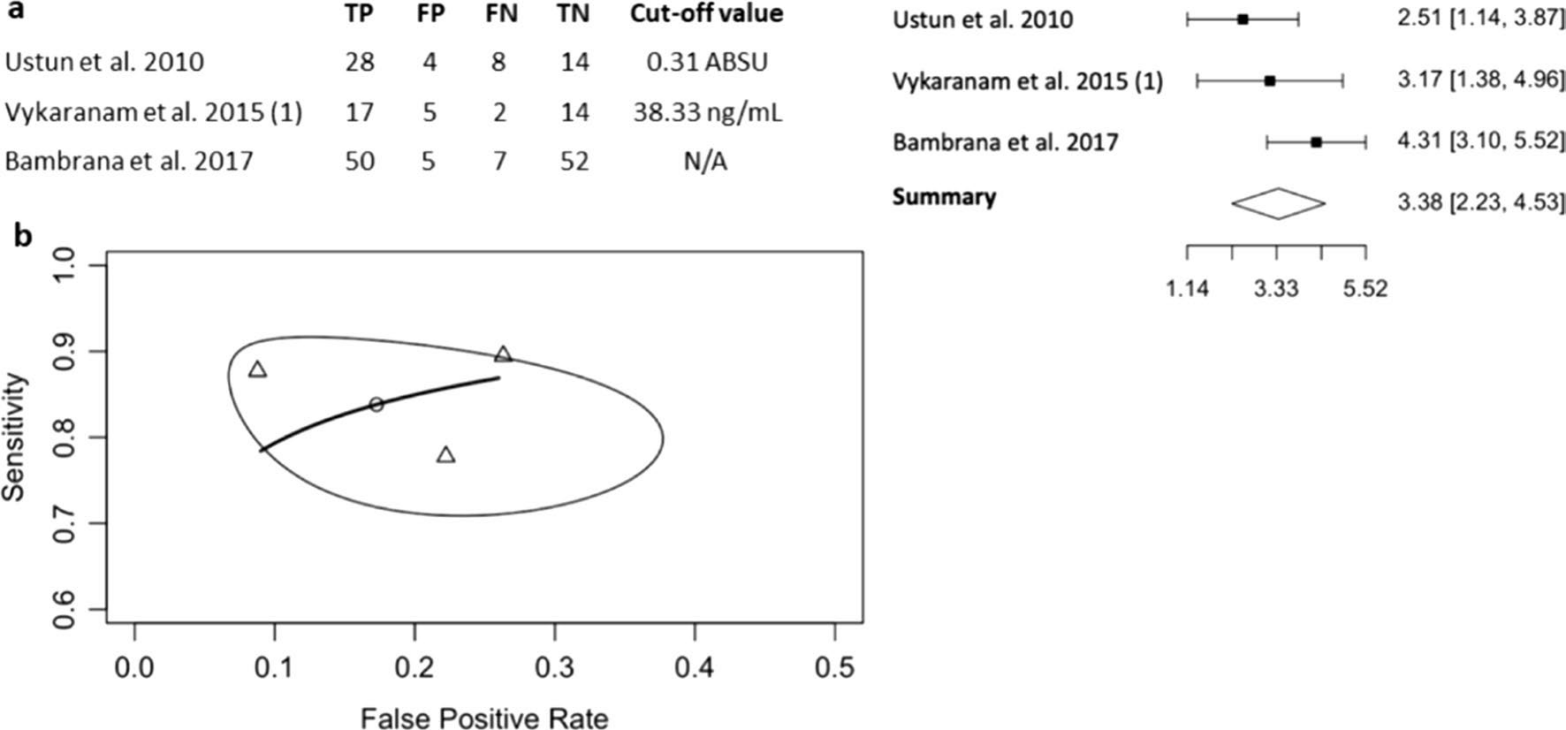
Diagnostic evaluation of IMA in preeclampsia using the bivariate, random-effects model. **a** Forest plot of three independent studies investigating the diagnostic performance of IMA in preeclampsia, with sensitivity and specificity reported and ln(DOR). **b** Plot of HSROC curve showing the estimated pooled diagnostic accuracy

Three studies measured IMA in preeclampsia [6, 13, 14], all of which reported serum IMA levels. IMA analyses were based on 112 women with preeclampsia and 94 normotensive controls. Optimal cut-off values were recorded differently as absorbance units (ABSU) or concentration in ng/mL. Despite the varied cut-off values of IMA, the DOR was reliable (ln (DOR) 3.38, 95% CI 2.23, 4.53) and exhibited no heterogeneity (Higgins’ *I*^2^ = 0%, Cochran’s *Q* = 1.792, *p* = 0.408) (Fig. 2a). The pooled PPV of 0.852 (95% CI 0.728, 0.929) and NPV of 0.811 (95% CI 0.683, 0.890) were well-balanced (Table 2), and the pooled area under the curve (AUC) was 0.887 (Fig. 2b). All of these findings demonstrated the reliable diagnostic value of serum IMA as a marker for preeclampsia.

**Table 2:** Individual and pooled PPVs and NPVs of oxidative stress markers for preeclampsia

Study	TP	FP	FN	TN	PPV	NPV
IMA						
Ustun et al. [13]	28	4	8	14	0.875	0.636
Vykaranam et al. [14]	17	5	2	14	0.773	0.875
Bambrana et al. [6]	50	5	7	52	0.909	0.881
Pooled data					0.852	0.811
MDA						
Rani et al. [22]	26	4	4	26	0.867	0.867
Bambrana et al. [6]	48	18	9	39	0.727	0.813
Shaker et al. [23]	19	13	6	12	0.594	0.667
Pooled data					0.728	0.802
UA						
Nikolic et al. [3]	26	13	6	47	0.667	0.887
Bambrana et al. [6]	41	6	16	51	0.872	0.761
Vykaranam et al. [15]	26	5	4	26	0.839	0.867
Pooled data					0.795	0.833

**Fig. 3:**
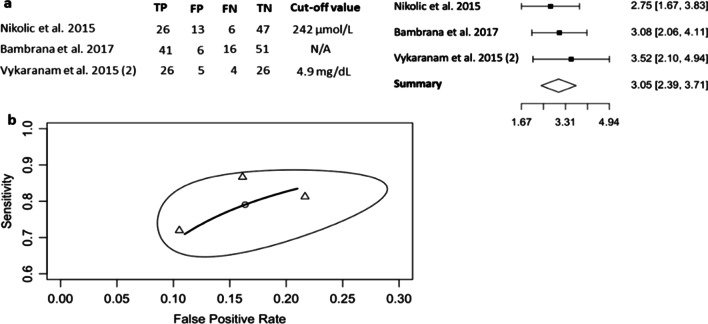
Diagnostic evaluation of UA in preeclampsia using the bivariate, random-effects model. **a** Forest plot of three independent studies investigating the diagnostic performance of UA in preeclampsia, with sensitivity and specificity, reported and ln(DOR). **b** Plot of HSROC curve showing the estimated pooled diagnostic accuracy

Three studies evaluating UA in preeclampsia were included in the meta-analysis [3, 6, 15], all of which determined serum UA levels. UA analyses were based on a pooled sample of 119 women with preeclampsia and 148 healthy controls. The optimal cut-off values were documented as µmol/L or mg/dL. Irrespective of the varied cut-off points for UA, the DOR was consistent (ln(DOR) 3.05, 95% CI 2.39, 3.71) and demonstrated no heterogeneity (Higgins’ *I*^2^ = 0%, Cochran’s *Q* = 0.717, *p* = 0.699) (Fig. 3a). The pooled PPV of 0.795 (95% CI 0.677, 0.877), NPV of 0.833 (95% CI 0.741, 0.897), were consistent between studies (Table 2). The pooled AUC was 0.881 (Fig. 3b). All of these findings demonstrated the reliable diagnostic value of serum UA as a biomarker for preeclampsia.

**Fig. 4:**
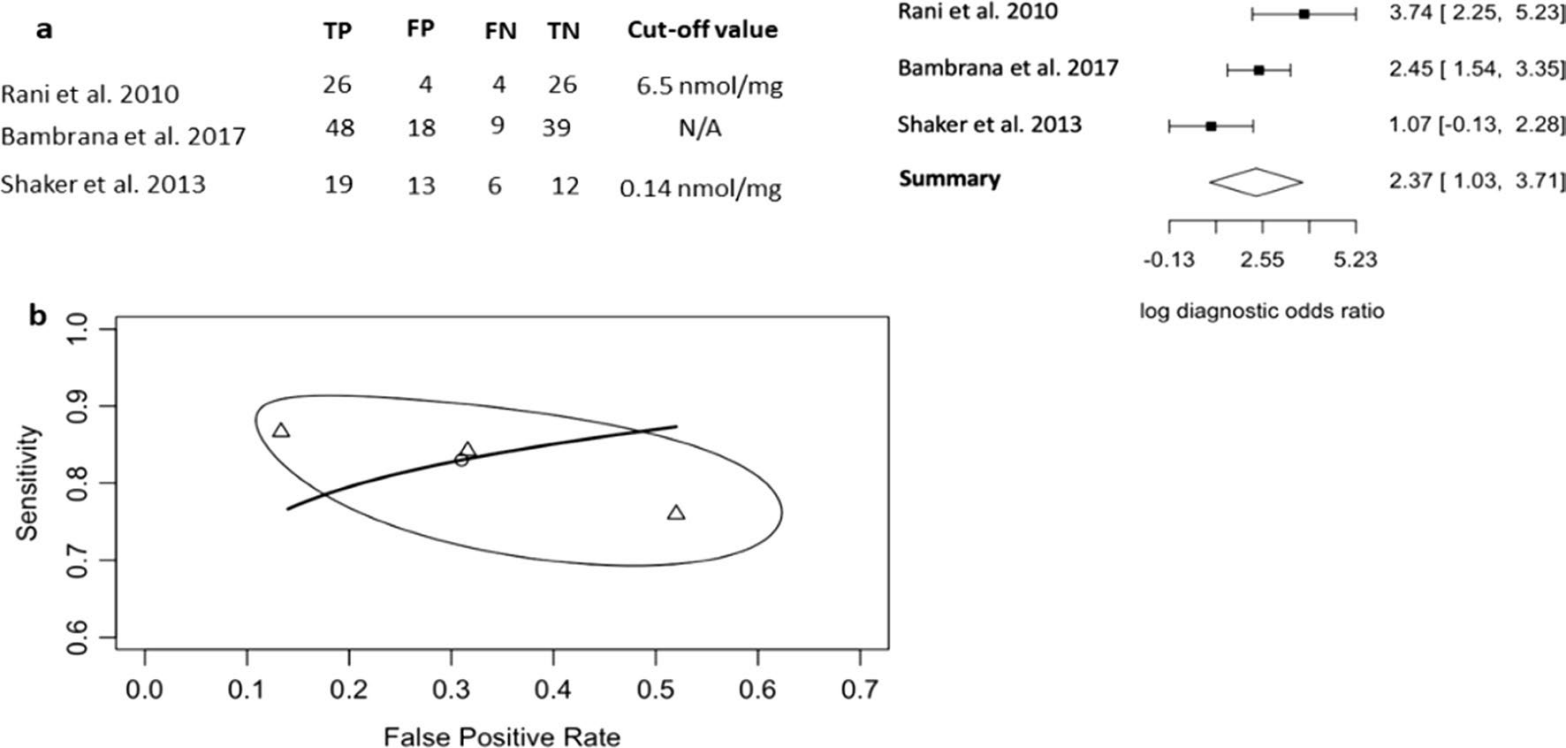
Diagnostic evaluation of MDA in preeclampsia using the bivariate, random-effects model. **a** Forest plot of three independent studies investigating the diagnostic performance of MDA in preeclampsia, with sensitivity and specificity reported and ln(DOR). **b** Plot of HSROC curve showing the estimated pooled diagnostic accuracy

MDA was evaluated using three studies following preeclampsia diagnosis or delivery of the baby [6, 22, 23]; two reported MDA concentrations from placental lysates using immunoblotting or ELISA and one reported serum concentration. The meta-analysis of MDA was based on a sample set of 112 women with preeclampsia and 112 normotensive controls. The optimal cut-off values were recorded as nmol/g or nmol/mg. Here, the DOR of MDA appear reliable (ln(DOR) 2.37, 95% CI 1.03, 3.71) and had low heterogeneity (Higgins’ *I*^2^ = 15.879%, Cochran’s *Q* = 2.378, *p* = 0.305) (Fig. 4a), which is acceptable as it is within 0–25% [32]. The pooled PPV of 0.728 (95% CI 0.564, 0.869), NPV of 0.802 (95% CI 0.615, 0.897), respectively, were consistent between the studies (Table 2).The pooled AUC was 0.845 (Fig. 4b). All of these findings demonstrated the reliable diagnostic value of MDA as a diagnostic biomarker for preeclampsia.

### Trace metals

Although there were three independent studies evaluating three trace metals (Cu, Zn, and Se) identified in our search, we were unable to pool the studies for each trace metal for the purpose of performing meta-analyses due to substantial heterogeneity between the studies. One such factor is that the direction of the cut-off value (higher or lower) is not consistent for any of the metals, and in some studies, these were not reported. However, many of the values used are within the normal reference range [5, 11]. Regarding Zn and Cu, studies reported inconsistent higher and lower cut-off values that were highly variable across the identified studies [5, 16, 19, 26]. Previous research showed inconsistent results for trace metals (Cu, Zn, and Se) in studies that included women with preeclampsia compared to healthy pregnancy [7, 16, 18, 19, 27]. The vast majority of studies [7, 33] showed that Cu, Zn, and Se were significantly decreased in women with preeclampsia compared with healthy controls. However, some reported increased levels [16, 19, 26]. Although there was no statistically significant difference, a trend towards reduced Se concentration in plasma was reported in one study [7]. El-Dorf et al. [11] also reported that increased placental concentration of Se and Zn, within the safe recommended range, were associated with improved fertility and decreased clinical risk of preeclampsia. Another study reported that Zn remained unchanged in preeclampsia compared to healthy controls (*p* = 0.724), although there was a positive correlation between serum Zn concentration in preeclampsia and diastolic blood pressure (> 100 mmHg) [5]. Furthermore, higher and lower abundance of Cu and Zn, respectively, were reported in women with preeclampsia compared to women with healthy pregnancies or gestational hypertension [19]. However, women with preeclampsia demonstrated significantly higher systolic blood pressure compared to gestational hypertension [19]. In terms of Se, lower serum concentration was present in preeclampsia compared to normotensive controls and there was a significant negative correlation between Se and systolic or diastolic blood pressure, or severity of preeclampsiay [7]. Overall trace metals showed different trends in published studies and conflicting results for the diagnosis of preeclampsia (Additional file 1: Table S1).

## Discussion

The pathogenesis of preeclampsia is still not fully understood, which has impeded the development of clinically useful diagnostic biomarkers, particularly those of evolving preeclampsia. Given the importance of oxidative stress in preeclampsia, this meta-analysis evaluated the diagnostic accuracy of the most promising oxidative stress markers in preeclampsia. After removing duplicates, out of 978 articles identified through our search, only nine studies met the prespecified inclusion criteria and were included in meta-analyses to evaluate the diagnostic potential of three oxidative stress biomarkers (IMA, UA, and MDA) for preeclampsia. In this meta-analysis, preeclampsia was not differentiated into its subtypes, such as early- and late-onset preeclampsia. In this unstratified preeclampsia group all three oxidative stress biomarkers, IMA, UA, and MDA, showed reliable parameters of diagnostic accuracy indicative of their diagnostic biomarker potential in preeclampsia. The values of DOR in the manuscript are all natural logarithm-transformed [ln(DOR)]. If transformed to the original value, all of these values are above the value of 10 hence with a reasonable diagnostic potential. These biomarkers also demonstrated satisfactory PPVs and NPVs for preeclampsia compared to healthy/normotensive pregnancies with AUCs above 0.84.

In addition to their biomarker potential, these oxidative stress molecular markers may also have important roles in the pathogenesis of preeclampsia. During early gestation in women who proceed to develop preeclampsia, inappropriate trophoblast cell invasion of spiral uterine arteries leads to oxidative stress in the placenta and increased formation of free radicals [34, 35]. All three identified oxidative stress biomarkers have been reported to be involved in abnormal placentation characterized by inadequate trophoblast invasion and remodeling of spiral uterine arteries observed in preeclampsia [28]. This includes IMA as a marker of ischemic events [6, 13, 14], UA as a marker of non-enzymatic antioxidants [3, 6, 15], and MDA as a marker of lipid peroxidation [6, 22, 23], which may have an important roles in the pathogenesis of preeclampsia.

Albumin is an important part of the antioxidant system and IMA formation is related to oxidative stress [14]. Within the hypoxic environment typical of the preeclamptic placenta, the N-terminal region of albumin has reduced capacity to bind to cobalt and this chemically changed albumin is referred to as IMA [14, 20]. Currently, IMA is utilized as an established marker for ischemic heart diseases and approved by the US Food and Drug Authority (FDA) as the first biomarker with specific clinical application [35–38]. Based on the degree of interaction between IMA and metal ions especially cobalt (Co^2+^), assays quantifying IMA known as the Albumin Cobalt Binding (ACB) test are used [35, 39–41]. This test measures IMA in human serum and it is reliable for the early detection of myocardial ischemia [40–42]. Importantly, the ACB test is sensitive to changes in IMA concentration (6–10 min after ischemia) that remain high for 6 h, which contributes to its reliable clinical utility [40]. Thus, IMA is also important in the accurate and timely diagnosis of different ischemia-related diseases [35, 39–41]. Given that frequently preeclampsia develops because of the placental ischemia that leads to endothelial and blood vessel damage [35], it is not surprising that IMA is an important player in preeclampsia with promising diagnostic biomarker potential. Several previous studies reported that overproduction of free radicals in the placenta from women with preeclampsia is related to ongoing ischemia [35, 36, 38, 43]. IMA is partly mediated by hypoxia/ischemia-driven oxidative stress that is elevated in women with preeclampsia [36]. Several previous studies showed that IMA levels were elevated in the early gestational stage in women who proceeded to develop preeclampsia, indicative of the presence of oxidative stress [13, 14]. Although we did not evaluate the predictive but rather diagnostic potential of IMA, in our meta-analysis and systematic review IMA was identified as one of the most promising biomarkers for the diagnosis of preeclampsia.

Conversely, UA has prooxidant function [44] and is a prominent marker of tissue injury and renal dysfunction [45, 46]. Apart from pregnancy, hyperuricemia has been associated with the onset of hypertension, cardiovascular and renal diseases [46]. Elevation of UA appears to occur concomitantly with high blood pressure and precedes the progression of proteinuria, which are the key features of preeclampsia [44, 46]. Several studies reported increased UA in early gestation in women who proceeded to develop preeclampsia [3, 6, 15, 44], suggesting its potentially useful predictive biomarker utility, which was not investigated in this meta-analysis. There are multiple sources of elevated UA in women with preeclampsia and it has been suggested that UA could be involved in its pathogenesis [46, 47]. Elevated UA affects placental and vascular health in pregnancy through the generation of oxidative stress and inflammation leading to preeclampsia [45–47]. Also, UA contributes to inappropriate vascular remodeling of the placental bed, which impedes trophoblast invasion [46]. Following this, the placenta can become ischemic, secreting various angiogenic factors leading to further endothelial cell damage and consequently preeclampsia [46]. Interestingly, if high UA is indeed linked to preeclampsia, a new FDA-approved medication, febuxostat, used for the treatment of the arthritic condition, gout, that reduces high UA levels [48], may be beneficial in preeclampsia. Relevant to this, a two-fold increased risk of rheumatoid arthritis, another form of arthritis, was observed in women with preeclampsia [49]. Normal UA plasma concentrations can vary based on sex and normal values for women are 1.5–6.0 mg/dL and for men are 2.5–7.0 mg/dL [50, 51]. UA is an important marker in diagnosing various conditions associated with higher levels including preeclampsia. In our meta-analysis, UA emerged as a useful biomarker for diagnosing preeclampsia based on the published pooled data [3, 6, 15], and the pathophysiology of preeclampsia is closely associated with increased abundance of UA in maternal–fetal/placental tissues in women with preeclampsia [44–47]. However, increased levels of UA (and creatinine) were reported in other hypertensive disorders of pregnancy questioning its specificity [15].

The potential of MDA as a biomarker of preeclampsia is reflective of the lipid peroxidation process within the plasma membranes of syncytiotrophoblast [22, 23]. Indeed, as a prominent marker of lipid peroxidation and oxidative damage, MDA expression correlates with the severity of preeclampsia [6, 23, 52]. Although the pathophysiology of preeclampsia is still poorly understood, many researchers propose that it involves endothelial cell damage induced by ROS that results in uncontrolled lipid peroxidation [53, 54]. Endothelial dysfunction is also responsible for the disruption of the nitric oxide pathway, decreased blood flow, and poor placentation [55]. The imbalance between free radicals and antioxidants is indicative of cell damage leading to oxidative stress and, subsequently, enhanced lipid peroxides in preeclampsia [55]. MDA is a three-carbon aldehyde and produces free radicals, which act on polyunsaturated fatty acids on the lipid bilayer. Due to the loss of fluidity and enhanced permeability to calcium ions and protons, lipid peroxidation reduces the membrane potential and breakdown of the cell membrane [55]. MDA is elevated in a number of diseases including pregnancy-induced hypertension and preeclampsia [54]. The methodology used for measuring MDA concentrations in human plasma is simple and robust [56]. Most studies use sensitive and reproducible high-performance liquid chromatography (HPLC) to determine the abundance of MDA in human plasma samples [56–58]. Here, the fluorescence-generating agent thiobarbituric acid (TBA) is used to bind MDA and the levels of MDA–TBA adduct are assessed [57]. Compared to conventional colorimetric analysis methods using HPLC for MDA–TBA adduct separation minimizes interference from TBA-reactive substances (TBARS) [57] and achieves good sensitivity and specificity for the analysis of MDA [55, 56]. As an established lipid peroxidation marker, it is logical that MDA could be utilized to aid the diagnosis of preeclampsia. Our robust meta-analysis confirmed its useful diagnostic utility, however other studies have also shown that increased plasma MDA produces free radicals that damage the cell membrane and correlate with disease severity [6, 22, 23, 52–54]. Normal plasma MDA concentrations are in the range of 1.40–1.90 nmol/dL outside of pregnancy settings; however, MDA plasma levels are generally elevated throughout pregnancy with further increases being observed in pregnancy with preeclampsia [56, 58]. In our meta-analysis, MDA was identified as an important biomarker for preeclampsia diagnosis and given that the pathophysiology of preeclampsia is closely related to aberrant lipid peroxidation and higher MDA concentration, it could also be explored as a therapeutic target.

### Limitations

In this study, we identified the most promising oxidative stress biomarkers for diagnosis not prediction of preeclampsia hence future studies should investigate their potential in predicting preeclampsia in asymptomatic patients. The longitudinal predictive studies are lacking and should be carried out in the future where plasma or serum samples are used to measure IMA, UA, MDA concentration pre- and post-diagnosis of preeclampsia. Although our systemic review reports interesting and robust results, some studies related to the MDA marker used placental samples to measure this oxidative biomarker concentration in addition to serum. Although the vast majority of studies collected patient samples before the onset of delivery, small percentage of the samples were collected after delivery, which needs to be taken into the account for future studies given that this can influence oxidative stress marker expression. We identified trace metals (Cu, Zn, and Se) as potentially important biomarkers of preeclampsia however due to unclear cut-off values, a meta-analysis could not be performed [5, 16, 19, 26]. Another limitation of this study is that all preeclampsia cases were grouped together without the acknowledgement of different phenotypes. It is important to assess the relevance of these biomarkers in specific phenotypes of preeclampsia and future research in this field should stratify participants according to preeclampsia phenotypes including early- and late-onset preeclampsia. The impact of fetal sex on circulating oxidative stress biomarkers in preeclampsia is another important factor however we were unable to obtain this information for the vast majority of our studies. Only two studies [15, 23] from our identified nine studies reported fetal sex in both normotensive and preeclampsia cases. Future research should take fetal sex into the account when interpreting the regulation of oxidative stress biomarkers in preeclampsia. Nevertheless, the studies included in the meta-analysis related to IMA, UA, and MDA were all consistent with low heterogeneity. Through our search, we also identified other oxidative stress biomarkers including total antioxidant status, total oxidant status, and oxidative stress index, however, the outcomes of these studies were not related to preeclampsia only but included other endpoints such as HELLP syndrome or neurological development [43, 59, 60]. Also, we were unable to obtain a sufficient number of studies (i.e., *n* < 3) for many biomarkers including total thiol, native thiol, and disulfide, which meant that we could not perform meta-analysis on these. Finally, as described above, studies evaluating several trace metals showed conflicting and inconsistent results.

### Perspectives and significance

In this meta-analysis and systematic review, IMA, UA, and MDA were identified as the most promising biomarkers associated with preeclampsia and are all linked to oxidative stress. This is an important contribution to the knowledge of preeclampsia pathophysiology and diagnosis that informs direction of future research with potential clinical utility of these oxidative stress proteins both in the diagnostic and treatment context of preeclampsia. Although still in the early stages of development, following more research in larger cohort studies, these biomarkers could be useful for the timely diagnosis of preeclampsia. Among these, IMA appears the most accurate, and it is reflective of tissue damage or ischemia, which is well-known to be present in or lead to preeclampsia. This study focused on the diagnostic potential of these biomarkers however, they could also play important roles in the pathogenesis of preeclampsia based on their well-known involvement in aberrant placentation and hence be used for prediction of preeclampsia. Further studies should be conducted to investigate their biomarker and pathogenic roles to definitively identify their utility as important diagnostic tools and/or therapeutic targets for preeclampsia. In the future, these biomarkers could be utilized for timely diagnosis and prevention of complications and death associated with preeclampsia [10, 23]. Further evaluation of IMA, MDA and UA in larger cohorts of women with preeclampsia is needed towards clinical application.

## Conclusions

Preeclampsia is a multifactorial hypertensive disorder of pregnancy with pathogenic mechanisms that are still not fully understood. Oxidative stress has been shown to play one of the key roles in the pathogenesis of preeclampsia. In our meta-analysis, we identified three reliable oxidative stress biomarkers based on their diagnostic sensitivities and specificities. These biomarkers should be explored in larger studies for their diagnostic but also predictive and therapeutic target potential. Diagnosis of evolving preeclampsia can be challenging and hence these biomarkers could provide further assurances in addition to typical clinical characteristics of preeclampsia. Timely diagnosis and management of preeclampsia are key in reducing the risk of maternal and fetal death as well as other pregnancy complications including pre-term birth.

## Supplementary Information

**Additional file 1: Table S1.** Study characteristics of trace metals and other oxidative stress markers.

## Data Availability

The datasets used and/or analyzed during the current study are available from the corresponding author on reasonable request.
