# Supplementary material for: The diagnostic potential of oxidative stress biomarkers for preeclampsia: systematic review and meta-analysis
Source: Biol Sex Differ. 2022 Jun 4;13:26. doi: 10.1186/s13293-022-00436-0 (PMC9167545; doi:10.1186/s13293-022-00436-0)
Supplement: Supplementary file 1 — Additional file 1: Table S1. Study characteristics of trace metals and other oxidative stress markers. [file 13293_2022_436_MOESM1_ESM.docx]

**Additional Table S1: Study characteristics of trace metals and other oxidative stress markers**

| Oxidative stress (OS) markers | Author/  Reference | | | Study design | | Sample | | Optimal cut-off | | Control type | | N  (Control) | | PE type | | n (PE) | | TP | | FP | | FN | | TN | | Exclusion criteria |
| --- | --- | --- | --- | --- | --- | --- | --- | --- | --- | --- | --- | --- | --- | --- | --- | --- | --- | --- | --- | --- | --- | --- | --- | --- | --- | --- |
| Selenium (Se) | Wilson et al. 2018 [16] | | | Cohort | | Plasma | | <0.86 μmol/L | | GH | | 108 | | Mixed EOPE and LOPE | | 85 | | 33 | | 59 | | 52 | | 49 | | Pre-existing medical condition or obstetric history including a high risk of developing PE, sPTB, or a small-for-gestational age (SGA) infant was delivered |
|  | El-Dorf et al. 2019 [11] | | | Cohort | | Urine | | ≤35.4 (unit not known) | | Normotensive | | 390 | | Mixed EOPE and LOPE | | 30 | | 25 | | 146 | | 5 | | 244 | | Multiple pregnancies and fetal anomalies |
|  | Eze et al. 2020 [7] | | | Case control | | Serum | | <0.8 μmol/l | | Normotensive | | 58 | | Mixed EOPE and LOPE | | 58 | | 33 | | 10 | | 25 | | 48 | | HIV infection, chronic renal disease, diabetes mellitus, multiple gestations and those on medications that could impact on their Selenium levels e.g, supplements containing Selenium. Smoker and those drinking alcohol were also excluded. |
|  | | Mahomed et al. 2000 [19] | Case control | | Leukocyte | | >4.01 µg/g total proteins | | Normotensive | | 184 | | Mixed EOPE and LOPE | | 171 | | 56 | | 46 | | 115 | | 138 | | Chronic hypertension diagnosed prior to pregnancy or during the first 20 weeks of the index gestation | |
| Zinc (Zn) | | Ahsan et al. 2010 [5] | Case control | | Serum | | <0.35 µmol/L | | Normotensive | | 35 | | EOPE | | 45 | | 15 | | 12 | | 30 | | 23 | | Pre-existing hypertension and proteinuria before conception or before 20 weeks of gestation, any associated medical conditions, multiple gestations | |
|  |  | Wilson et al. 2018 [16] | Cohort | | Plasma | | <8.3 μmol/L | | GH | | 108 | | Mixed EOPE and LOPE | | 85 | | 26 | | 42 | | 59 | | 66 | | Pre-existing medical condition or previous obstetric complication predisposing to PE, sPTB or a small-for-gestational age (SGA) | |
|  |  | Mahomed et al. 2000 [19] | Case control | | Leukocyte | | >183.5 µg/g total proteins | | Normotensive | | 184 | | Mixed EOPE and LOPE | | 171 | | 78 | | 46 | | 93 | | 138 | | Chronic hypertension diagnosed prior to pregnancy or during the first 20 weeks of the index gestation | |
| Copper (Cu) | | Gul et al. 2021 [26] | Case control | | Serum | | 224 μg/dL (=35.26 μmol/L) | | Normotensive | | 45 | | Mixed EOPE and LOPE | | 43 | | 25 | | 6 | | 18 | | 39 | | Diabetes mellitus, obesity (body mass index > 30 kg/m2), chronic disorders before pregnancy, drug/alcohol/cigarette use, genetic disorders, current history of surgery or blood transfusion, any medications, fetal anomalies, fetal death in utero, active infection | |
|  |  | Wilson et al. 2018 [16] | Cohort | | Plasma | | 32.5 μmol/L | | GH | | 108 | | Mixed EOPE and LOPE | | 85 | | 37 | | 39 | | 48 | | 69 | | Pre-existing medical condition or obstetric history predisposing to high risk of developing PE, sPTB or a small-for-gestational age (SGA) infant delivered | |
|  |  | Mahomed et al. 2000 [19] | Case control | | Leukocyte | | >17.0 µg/g total proteins | | Normotensive | | 184 | | Mixed EOPE and LOPE | | 171 | | 89 | | 92 | | 82 | | 92 | | Chronic hypertension diagnosed prior to pregnancy or during the first 20 weeks of the index gestation | |
| Disulfide (S-S) | | Onat et al. 2020 [20] | Case control | | Whole blood | | 29.05 µmol/L | | Normotensive | | 57 | | Mixed EOPE and LOPE | | 47 | | 31 | | 16 | | 16 | | 41 | | History of PE, chronic gestational hypertension, renal, hepatic and thyroid disorders, type I and type II diabetes mellitus | |
|  |  | Yuvaci et al. 2016 [21] | Case control | | Whole blood | | ⩽13.05 µmol/L | | Normotensive | | 37 | | Severe PE | | 32 | | 20 | | 13 | | 12 | | 24 | | History of PE, pre-existing chronic diseases, use of drugs affecting renal and liver functions, chronic hypertension, gestational diabetes mellitus, type I/type II diabetes mellitus, connective tissue diseases, chronic renal and liver diseases, hyper/hypothyroidism, hematologic diseases, chromosomal/congenital fetal anomaly disorder, multiple pregnancies, and identified urine infection | |
| Native thiol (-SH) | | Onat et al. 2020 [20] | Case control | | Whole blood | | 178.45 µmol/L | | Normotensive | | 57 | | Mixed EOPE and LOPE | | 47 | | 34 | | 10 | | 13 | | 47 | | History of PE, chronic gestational hypertension, renal, hepatic and thyroid diseases, type I and type II diabetes mellitus | |
|  |  | Yuvaci et al. 2016 [21] | Case control | | Whole blood | | ≤276.9 µmol/L | | Normotensive | | 37 | | Severe PE | | 32 | | 31 | | 14 | | 1 | | 23 | | History of PE, pre-existing chronic diseases, use of drugs affecting renal and liver functions, chronic hypertension, gestational diabetes mellitus, type I/type II diabetes mellitus, connective tissue diseases, chronic renal and liver diseases, hyper/hypothyroidism, hematologic diseases, chromosomal/congenital fetal anomaly disorders, multiple pregnancies, and identified urine infection | |
| Total thiol (-SS+-SH) | | Onat et al. 2020 [20] | Case control | | Whole blood | | 232.55 µmol/L | | Normotensive | | 57 | | Mixed EOPE and LOPE | | 47 | | 35 | | 9 | | 12 | | 48 | | History of PE, chronic or gestational hypertension, renal, hepatic and thyroid diseases, type I and type II diabetes mellitus | |
|  |  | Yuvaci et al. 2016 [21] | Case control | | Whole blood | | 311.6 µmol/L | | Normotensive | | 37 | | Severe PE | | 32 | | 32 | | 15 | | 0 | | 22 | | History of PE, pre-existing chronic diseases, use of drugs affecting renal and liver functions, chronic hypertension, gestational diabetes mellitus, type I/type II diabetes mellitus, connective tissue diseases, chronic renal and liver diseases, hyper/hypothyroidism, hematologic diseases, chromosomal/congenital fetal anomaly disorders, multiple pregnancies, and identified urine infection | |
| Stronium (Sr) | | Barneo-Caragol et al. 2019 [17] | Case control | | Serum | | 29.4 μg/L | | IUGR/GH, gestational week>34 week | | 40 | | EOPE | | 28 | | 22 | | 12 | | 6 | | 28 | | Multiple pregnancies were excluded | |
|  |  | Barneo-Caragol et al. 2018 [18] | Case control | | Serum | | 31.3 μg/L | | Normotensive | | 104 | | EOPE | | 39 | | 29 | | 36 | | 10 | | 68 | | Multiple pregnancies and samples collected post-partum | |

**PE: preeclampsia; EOPE: early onset preeclampsia; LOPE: late onset preeclampsia; TP: true positive; FP: false positive; TN: true negative; FN: false negative; HELLP syndrome: (haemolysis, elevated liver enzymes, and low platelet count) syndrome; IUGR: intrauterine growth restriction; GH: gestational hypertension; sPTB: pre-term birth**
